# Antiepileptic Drugs during Pregnancy in Primary Care: A UK Population Based Study

**DOI:** 10.1371/journal.pone.0052339

**Published:** 2012-12-18

**Authors:** Shuk-Li Man, Irene Petersen, Mary Thompson, Irwin Nazareth

**Affiliations:** 1 Research Department of Primary Care and Population Health, University College London, London, United Kingdom; 2 Cegedim Strategic Data Medical Research UK, London, United Kingdom; Federal University of Rio de Janeiro, Brazil

## Abstract

**Objective:**

Antiepileptic drugs (AEDs) are commonly prescribed for epilepsy and bipolar disorder but little is known about their use in pregnancy. We examined secular trends in AED prescribing in pregnancy and pregnancy as a determinant for stopping AED prescribing.

**Methods:**

We identified 174,055 pregnancies from The Health Improvement Network UK primary care database. Secular trends in AED prescribing during pregnancy were examined between 1994 and 2009. We used Cox's regression analyses to compare time to discontinuation of AED prescriptions between pregnant and non-pregnant women and to identify predictors of discontinuation of AEDs in pregnancy.

**Results:**

Prescribing of carbamazepine and sodium valproate have declined since 1994 despite being the most commonly prescribed AEDs in pregnancy up to 2004. Prescribing of lamotrigine in pregnancy has steadily increased and has been the most popular AED prescribed in pregnancy since 2004. Pregnant women with epilepsy were twice as likely to stop receiving AEDs (Hazard Ratio (HR) 2.00, 95% Confidence Interval (CI) 1.62–2.47) when compared to non-pregnant women and for women with bipolar disorder this was even higher (HR 3.07, 95% CI 2.04–4.62). For pregnant women with epilepsy, those receiving AEDs less regularly before pregnancy were more likely to stop receiving AEDs in pregnancy.

**Conclusions:**

Lamotrigine has been increasingly prescribed in pregnancy over older AEDs namely carbamazepine and sodium valproate. Pregnancy is a strong determinant for the discontinuation of AED prescribing particularly for women with bipolar disorder.

## Introduction

In pregnancy, some older antiepileptic drugs (AEDs) have been found to increase the risk of major congenital malformations (MCMs), and in particular, sodium valproate is also associated with developmental delay [1], [2]. The benefit of maintaining AEDs in pregnancy for most women is to control seizures that can harm the fetus and mother [3]. Over a third of women with bipolar disorder are also treated with AEDs, [4] which if untreated in pregnancy, increases the risk of mood episode relapse and postpartum psychosis [5]–[7]. This leaves women taking AEDs and their healthcare professionals with a dilemma as to whether to continue taking AEDs in pregnancy.

The 2012 clinical guidelines from the National Institute of Health and Clinical Excellence (NICE) advised caution on the use of sodium valproate in pregnancy, but their guidance offers little other advice on which AEDs are safe to use in pregnancy. However, they recommend seizure freedom in pregnancy should be sought amongst women with epilepsy [3]. The British National Formulary (BNF) also emphasises the benefit of continuation of therapy in pregnancy, stating that the “risk of harm to the mother and fetus from a convulsive seizure outweighs that of continued therapy” [8].

There are limited data on how prescribing of AEDs in pregnant women has changed over time. Evidence based guidance suggest that changes to a woman's treatment regimen should be made in the planning stages of pregnancy to eliminate the need to stop abruptly, or to switch AEDs during pregnancy [3]. We examined secular trends of prescribing common AEDs in pregnancy and assessed whether pregnancy is a major determinant for the discontinuation of AED prescribing.

**Figure 1:**
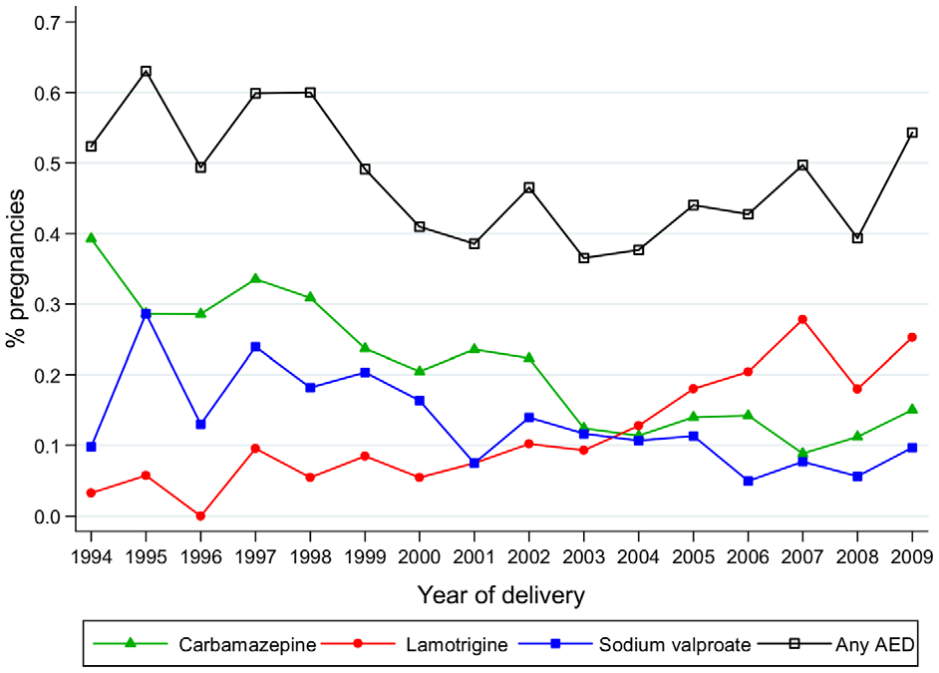
Percentage of pregnancies where AEDs were prescribed.

## Methods

### Study design

We conducted a cohort study of pregnant women taking AEDs using data from The Health Improvement Network (THIN) (http://csdmruk.cegedim.com/). This is one of the UK's largest primary care databases, containing anonymised records for over 9 million patients in 480 practices and is broadly representative of the UK population in terms of sex, age, size of practice and geographic distribution [9]. It records the data collected during a patient's visit to their general practitioner (GP), including medical diagnoses and symptoms (based on the Read code system), [10] additional health data (such as smoking status, test results and pregnancy details), prescriptions, referrals to secondary care and anonymised free text information. Demographic information such as the patient's date of birth and sex, and a marker of social deprivation, the Townsend quintile, [11] derived from 2001 census data, are also included.

**Table 1:** Factors associated with discontinuation of AEDs amongst pregnant women.

	Pregnant women with epilepsy (N = 745)					Pregnant women with bipolar disorder (N = 54)				
		Unadjusted		Adjusted			Unadjusted		Adjusted	
	N	HR (95% CI)	p-value	HR (95% CI)	p-value	N	HR (95% CI)	p-value	HR (95% CI)	p-value
Age (years)			0.148		0.282			0.031		0.100
<25	184	1.26 (0.97, 1.63)		1.24 (0.95, 1.63)		5	1.24 (0.48, 3.25)		1.23 (0.39, 3.91)	
25–34	440	1		1		33	1		1	
35+	121	0.94 (0.68, 1.30)		1.01 (0.73, 1.40)		16	0.40 (0.19, 0.82)		0.42 (0.18, 0.95)	
Depression/ bipolar disorder			0.167		0.732					
No	662	1		1			n/a		n/a	
Yes	83	1.26 (0.91, 1.76)		1.06 (0.75, 1.52)						
Townsend			0.045		0.208			0.154		0.266
1	136	1		1		9	1		1	
2	113	0.92 (0.62, 1.38)		0.99 (0.66, 1.49)		7	0.68 (0.22, 2.08)		0.74 (0.20, 2.76)	
3	141	0.93 (0.63, 1.36)		0.94 (0.63, 1.38)		9	1.40 (0.52, 3.77)		1.43 (0.45, 4.51)	
4	177	1.39 (0.99, 1.96)		1.17 (0.82, 1.67)		12	2.04 (0.83, 5.03)		2.02 (0.75, 5.48)	
5	130	0.90 (0.61, 1.34)		0.76 (0.50, 1.15)		11	0.78 (0. 29,2.09)		0.85 (0.28, 2.58)	
Missing	48	1.39 (0.87, 2.24)		1.30 (0.80, 2.11)		6	1.05 (0.34, 3.23)		1.67 (0.48, 5.83)	
Previous AEDs			<0.0001		<0.0001			0.159		0.381
0	70	6.47 (4.71, 8.88)		6.32 (4.57, 8.73)		15	1.92 (0.98, 3.77)		1.52 (0.66, 3.50)	
1	204	3.31 (2.58, 4.24)		3.30 (2.57, 4.23)		10	1.45 (0.68, 3.07)		0.74 (0.30, 1.79)	
2+	471	1		1		29	1		1	
Co-medications					0.389			0.269		0.148
0	583	1	0.385	1		7	2.01 (0.85, 4.78)		2.63 (0.99, 7.03)	
1	119	1.20 (0.89, 1.61)		1.22 (0.90, 1.64)		18	1.32 (0.69, 2.51)		1.39 (0.71, 2.72)	
2+	43	1.20 (0.77, 1.87)		1.20 (0.74, 1.92)		29	1		1	

For all variables, except Townsend, the most common category formed the reference group.

THIN was approved for use in scientific research by the National Health Service South-East Multi-centre Research Ethics Committee in 2003. This study was further approved by the Scientific Review Committee in 2011. Individual patient consent was not required because the data are anonymised at source.

**Figure 2:**
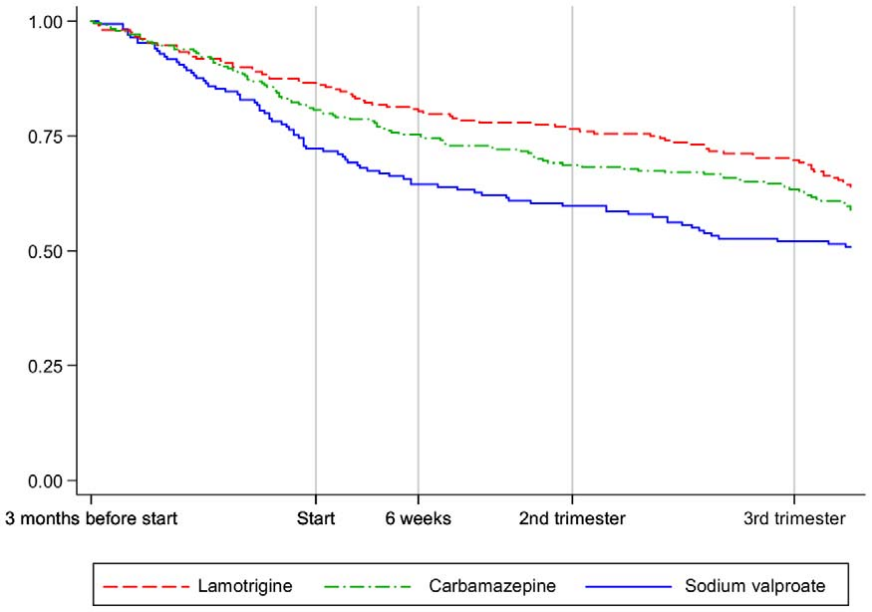
Proportion of pregnant women with epilepsy continuing AEDs by AED.

We identified 350,630 pregnancies and randomly selected one pregnancy per woman therefore including 174,055 pregnancies in this study. Women were first included if they were pregnant between 1994 and 2009 and their records met pre-defined standards for acceptable data recording [12].

**Figure 3:**
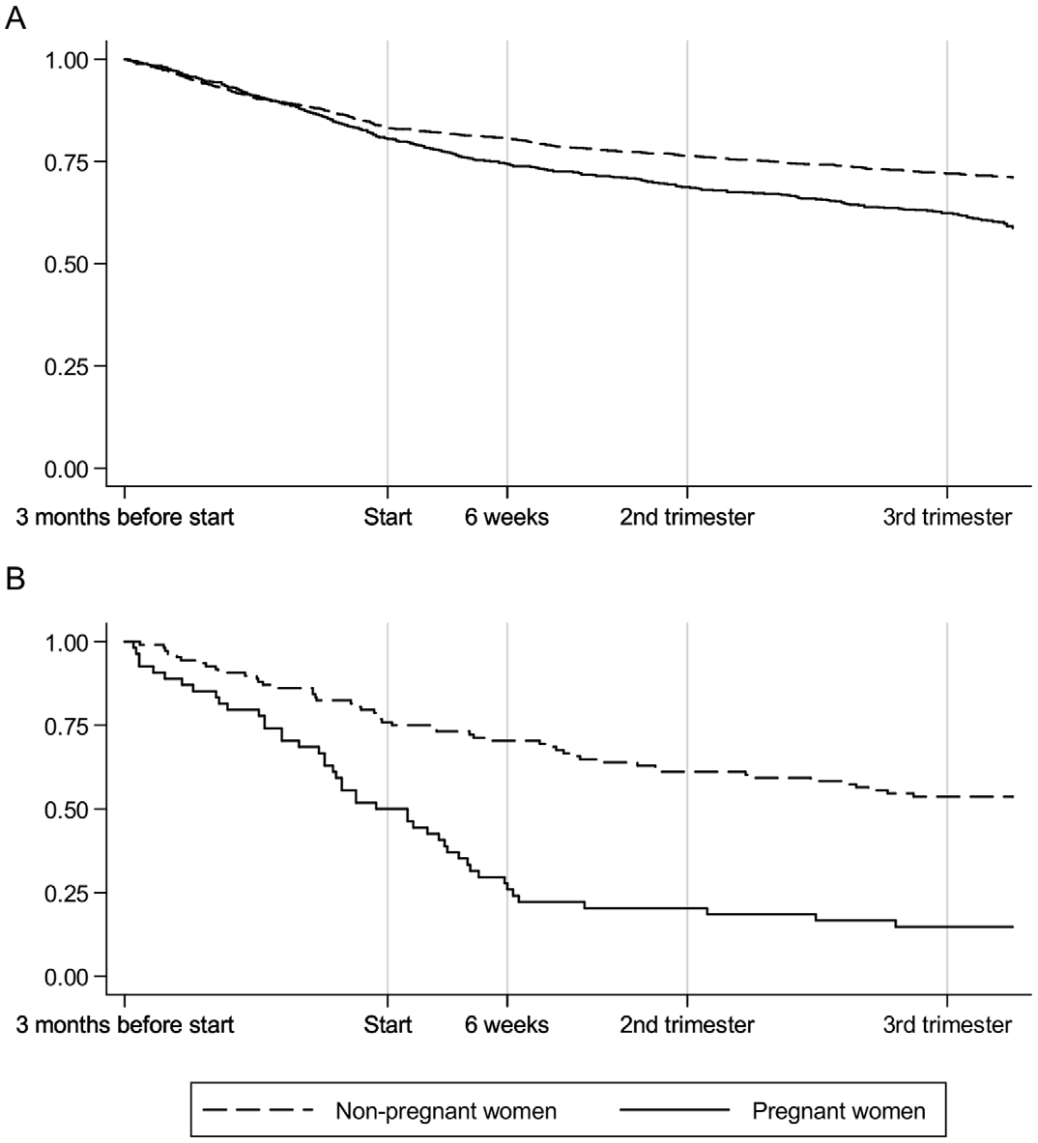
Proportion of women continuing AEDs: pregnant vs. non-pregnant women with A. epilepsy B. bipolar disorder or depression.

Pregnancy was defined by Read codes which indicated a delivery. The start of each pregnancy was determined by using the date of the last menstrual period, gestational age at birth, information on preterm delivery and associated free text data. Where this information was not available, the start of pregnancy was assumed to be 280 days before the delivery date. For the study of secular trends in prescribing, the women were required to be registered with the practice throughout their pregnancy and to have received more than one AED prescription within a three month period in pregnancy. For the study of discontinuation of AEDs, women were also required to be registered throughout the six months before pregnancy in order to identify those who were prescribed AEDs before pregnancy.

A group of non-pregnant women receiving AEDs was defined for comparison with pregnant women. This included women who had never been pregnant as well as women who had had one or more pregnancies. For the latter group we excluded periods where they were pregnant and excluded periods from two years before pregnancy to one year after a delivery. These periods were designed to exclude the time where planning pregnancy, pregnancy itself or breastfeeding may have an impact on drug treatment. One non-pregnant period per woman was chosen at random. A random index date was assigned in the non-pregnant period. Non-pregnant women were stratified by indication for AEDs and randomly selected within five year age bands so that the age distribution was similar to that of pregnant women. Two for every one pregnant woman taking AEDs were selected.

A list of encrypted Multilex ID codes relating to the AEDs listed in Chapter 4.8.1 of the BNF was created to identify AED prescriptions in THIN [8]. THIN does not automatically link a patient's prescriptions with the indication, therefore we used Read codes to identify women with the main indications for AEDs (epilepsy, bipolar disorder and depression) and searched for diagnoses made before the delivery date. Where none of these diagnoses were found, medical records were interrogated to find plausible indications. Both drug and Read code lists were reviewed by a GP (IN).

### Data analyses

The percentage of pregnancies where AEDs were prescribed more than once was calculated by year of delivery.

Analysis of discontinuation of AED prescribing was performed on three groups – women with epilepsy, women with bipolar disorder or depression, and women with no or other indication for AEDs. For inclusion in the study, pregnant women had to be prescribed AEDs at least once in the three months before the start of pregnancy and non-pregnant women prescribed at least once in the three months preceding the index date. Their last consecutive AED prescription was determined if no other AEDs were prescribed within the subsequent three months of the previous prescription. Follow-up was measured from three months before the pregnancy start date and ended at the earlier of the last prescription date, delivery date if the birth was premature, or two months before the delivery date if the birth was full-term. For non-pregnant women, follow-up started three months before the index date and ended at the earlier of the last prescription date or 280 days after the index date. Cox's proportional hazards regression model was used to estimate hazard ratios (HRs) comparing the time to last prescription between pregnant and non-pregnant women, stratified by indication for AEDs. The proportion of women continuing to receive AED prescriptions is described at 92 days follow-up (i.e. ∼ start of pregnancy) and at 288 days (i.e. ∼ beginning of the third trimester). Amongst the women with no or other indication for AEDs, it was not possible to select a similar non-pregnant group of women, thus HRs were not estimated for this group.

Factors associated with the discontinuation of AED prescribing in pregnancy were analysed using Cox's regression amongst pregnant women with epilepsy and pregnant women with bipolar disorder or depression. Maternal age was categorised as younger than 25, 25–34 and 35+ years. Social deprivation was measured using the Townsend quintile, a postcode based indicator ranging from 1 for the least deprived to 5 for most deprived areas. The number of times AEDs were prescribed prior to the initiation of follow-up, i.e. in the three to six months before pregnancy, was counted and categorised as 0, 1 or 2+. Co-medication was measured as the number of different types of drugs prescribed for treatment of conditions affecting the central nervous system (BNF Chapter 4), excluding AEDs and was categorised as 0, 1 and 2+. Amongst women with epilepsy, co-morbidity with bipolar disorder or depression was also analysed as a risk factor. Univariable analyses of each of these factors and adjusted analyses including all factors in the regression model were performed. Separately, we also compared discontinuation of AEDs amongst pregnant women by specific AEDs prescribed in the three months before pregnancy.

Data were analysed using Stata 11.1.

## Results

### Secular trends in prescribing

Over the late 1990 s, approximately 0.5–0.6% of pregnancies had an AED prescribed during the course of the pregnancy. Over the period from 2000 to 2009, the rate fell slightly and varied between 0.37% in 2003 to 0.54% in 2009 (Figure 1). Up to 2004 carbamazepine was the most commonly prescribed AED in pregnancy followed by sodium valproate. Prescribing of carbamazepine and sodium valproate in pregnancy, however, fell by 70% from 1994 to 2004. The prescribing of lamotrigine in pregnancy steadily increased from 2000 onwards and since 2004 has been the most commonly prescribed AED in pregnancy. By 2009, 0.25% of pregnant women were prescribed lamotrigine in pregnancy; carbamazepine was prescribed in 0.15% of pregnancies and sodium valproate in 0.10%.

### Factors associated with discontinuation of AEDs in pregnancy

Overall, 934 pregnant women received AED prescriptions in the three months before pregnancy. Of these, 745 women had a clinical record of epilepsy and 54 of bipolar disorder or depression.

#### Women with epilepsy

The majority of the 745 women prescribed AEDs for epilepsy were aged 25–34 years, were prescribed AEDs more than once prior to follow-up and were receiving no other medication (Table 1).

The frequency of AED prescriptions prior to follow-up significantly impacted upon the time to last AED prescription in pregnant women with epilepsy (Table 1). Women with no prescriptions in the three to six months before pregnancy were six times more likely to have stopped receiving AEDs in pregnancy than those who had been prescribed on more than one occasion (HR 6.32, 95% CI 4.57–8.73), whilst women with just one prescription in the three to six months before pregnancy were three times more likely to stop receiving treatment in pregnancy (HR 3.31, 95% CI 2.57–4.23).

#### Women with bipolar disorder or depression

The majority of the 54 women who were prescribed AEDs for bipolar disorder or depression were aged 25–34 years and had been prescribed AEDs more than once during the three to six months before pregnancy. Most received two or more concomitant medications.

None of the examined factors were found to be associated with the time to last AED prescription amongst pregnant women with bipolar disorder or depression (Table 1).

### Specific AED associations with discontinuation of AEDs in pregnancy

Figure 2 shows the discontinuation rates for pregnant women with epilepsy by AED (sodium valproate, carbamazepine, lamotrigine). Compared to those receiving sodium valproate, women prescribed lamotrigine were less likely to stop AEDs in pregnancy (HR 0.63, 95% CI 0.46–0.86). Amongst pregnant women with bipolar disorder or depression, data were too few to analyse.

### Pregnancy – a determinant for discontinuing AEDs?

#### Women with epilepsy

In 745 pregnant women with epilepsy who had received an AED during the three months prior to pregnancy, 601 (80.7%) also received AEDs during pregnancy and 369 (62.4%) were prescribed AEDs in their last trimester. In comparison, of 1490 non-pregnant women with epilepsy, 1240 (83.2%) received AEDs after 92 days and 1073 (72.0%) were prescribed AEDs after 288 days (Figure 3a). The HR for pregnant women with epilepsy stopping AEDs during pregnancy compared to non-pregnant women was 2.00 (95% CI 1.62–2.47).

#### Women with bipolar disorder or depression

Pregnant women with bipolar disorder or depression were three times as likely to discontinue AED prescriptions compared to non-pregnant women (HR 3.07, 95% CI 2.04–4.62). In pregnancy, only half were still being prescribed AEDs (N = 27) and only 8 (14.8%) were prescribed AEDs in the last trimester. In comparison, of 108 non-pregnant women 82 (75.9%) continued to receive AEDs after 92 days and 58 (53.7%) past 288 days (Figure 3b).

We reviewed the prescription records after the date of the last AED prescription for all 54 women, and found 17 (31%) continued to be prescribed antidepressants or antipsychotics after stopping AEDs. However, 27 (50%) stopped altogether (though some restarted after the first trimester).

#### Women with other indications for AEDs than epilepsy and bipolar disorder

In total 135 women were prescribed AEDs in the three months before pregnancy without an indication of epilepsy, bipolar disorder or depression. Only 59 (43.7%) continued receiving AEDs in pregnancy and 19 (14.1%) were prescribed AEDs in the final trimester. Of the 59 women who continued prescriptions into pregnancy, 26 had clinical records for chronic or neuropathic pain, 15 had records relating to treatment for mental health including depression (diagnoses made more than a year before pregnancy), anxiety and personality disorders, three with migraines, two had suspected epilepsy and the remaining 13 indications could not be ascertained from the medical records. Furthermore, of the 19 women who were still prescribed at the end of the second trimester, six were treated for mental health problems, another six for chronic or neuropathic pain, two with suspected epilepsy, one with migraines and indications for four women could not be identified.

## Discussion

There has been a decline in prescribing of the older AEDs namely carbamazepine and sodium valproate since 1994 whereas prescribing of lamotrigine, a newer AED, has increased five-fold since 2000. Amongst pregnant women with epilepsy, those receiving prescriptions more often prior to pregnancy were more likely to continue receiving AED prescriptions during pregnancy. Furthermore, those receiving lamotrigine were less likely to stop in pregnancy compared to women prescribed sodium valproate. Pregnancy was a determinant for the discontinuation of AED prescribing, in particular for women with bipolar disorder or depression. The majority of pregnant women prescribed AEDs for indications other than epilepsy and bipolar disorders discontinued prescriptions by six weeks into pregnancy.

The secular changes in prescribing habits observed in this study were similar to those seen in other countries. A fall in the use of carbamazepine and sodium valproate and a rise in lamotrigine were observed in the Australian Register of Antiepileptic Drugs in Pregnancy, the European and International Registry of Antiepileptic Drugs in Pregnancy and the Neurodevelopmental Effects of Antiepileptic Drugs study groups [13]–[15]. Sodium valproate and carbamazepine have been linked to severe teratogenic effects when taken in pregnancy [16]–[22]. Initially their prescription in pregnancy decreased over time. However, it is noticeable that since 2004 prescribing of these drugs in pregnancy remained relatively constant and that by 2009 they were still the second and third most commonly prescribed AED in pregnancy. The reasons for increased prescribing of lamotrigine are unclear – no formal guidance has been issued indicating the safety of lamotrigine. Recent evidence suggests no greater risk of MCMs is associated with lamotrigine when compared to untreated pregnancies in women with epilepsy [20], [21]. However, one study does report an increased risk of isolated cleft palate/lip in lamotrigine exposed babies compared to the general population and the BNF states lamotrigine is associated with increased teratogenicity [8], [23]. The guidance from the NICE is inconsistent. It states lamotrigine should not be prescribed to pregnant women with bipolar disorder because of the risk of harm to the fetus, but such advice is not provided in the guidance for women with epilepsy [3], [24]. Our results on secular prescribing trends and discontinuation of specific AEDs in pregnancy suggest that many healthcare professionals are selectively prescribing lamotrigine. Further data are required on the risks and benefits of prescribing lamotrigine in pregnancy.

To our knowledge, no other study has examined the discontinuation of AEDs in pregnancy. Our study showed that pregnant women were more likely to stop receiving AED treatment compared to non-pregnant women also receiving AEDs. This behaviour has been reported for other medications prescribed to pregnant women as they were concerned about the effect the drugs would have on the fetus [25], [26]. Our data cannot explain the behaviour, as we are unable to determine if it was the GP, the woman or both who were choosing to stop AEDs. We can only observe that women in pregnancy stop AEDs sooner than when they are not pregnant. Women with epilepsy behaved differently from those with bipolar disorder or depression. We separated these two groups since we had observed a similar steep decline in antidepressant prescribing in pregnancy in a previous study [27]. Pregnant women with epilepsy were twice as likely not to receive AEDs whilst this was more than three-fold in pregnant women with bipolar disorder or depression. We found a third of the women who were prescribed AEDs for bipolar disorders continued to be prescribed other alternative drugs such as antidepressants and antipsychotics.

Women with epilepsy who received frequent prescriptions prior to pregnancy were less likely to stop these drugs in pregnancy. These women may be those with a more severe form of the disease which requires regular consultation with their GP. It may also be that women receiving frequent AED prescriptions are those that are more likely to adhere to their medication.

We found no significant associations for the discontinuation of AEDs amongst pregnant women with bipolar disorder but we were limited by a small sample size.

Stopping medication in pregnancy is a choice that women should make rather than being forced to continue treatment against their wishes. The issue is whether women are fully informed before they make this choice. A recent survey of women with epilepsy highlighted failures in women receiving appropriate pre-conception counselling on the risks AEDs pose to the unborn child, despite the recommendation by NICE for this to be conducted for all women with epilepsy of childbearing potential [3], [28]. This suggests that women could be making decisions to stop or continue AED therapy without fully understanding the risks and benefits of their actions.

Healthcare professionals need to keep up to date with the latest information on the risks of AEDs in pregnancy [3]. There is lack of information on the relative risks between AEDs – a common choice healthcare professionals and women have to make. Research efforts in this area must therefore continue and must be more robust in order for stronger inferences to be made and clearer guidance to be provided.

The main strength of our study is the large sample size of women taking AEDs. Although this study is restricted to primary care, it captures the prescribing patterns for women who attend secondary and tertiary care. Neurologists and psychiatrists most often initiate this drug treatment, but in the UK the prescribing is then passed on to GPs who are wholly responsible for long term prescribing. Therefore the trends observed in this study are representative of most pregnant women taking AEDs.

The major limitation of the study is the verification of adherence to prescribed treatment. However, a recent study of UK prescriptions dispensing showed that over 98% of AEDs prescribed in general practice were dispensed [29].

We found a shift in the prescribing of AEDs in pregnancy from older to newer ones, in particular there has been a five-fold increase in the prescribing of lamotrigine since 1994. Pregnancy was a strong factor for the cessation of AED prescriptions, particularly in women with bipolar disorder. There are risks and benefits associated with the (dis)continuation of AEDs in pregnancy and it is important these are balanced to allow women and healthcare professionals to make an informed decision on whether to continue treatment in pregnancy. Further research is urgently needed to firmly establish the safety of AEDs in pregnancy, particularly for the increasingly prescribed AED, lamotrigine.
